# Antimicrobial Potential of Defensin-Derived *γ*-Core Peptides of *Thinopyrum elongatum* (Host) D.R. Dewey as Bio-Inspired Pesticides

**DOI:** 10.3390/ijms27104219

**Published:** 2026-05-09

**Authors:** Marina P. Slezina, Tatyana I. Odintsova

**Affiliations:** Vavilov Institute of General Genetics Russian Academy of Sciences, 119333 Moscow, Russia; omey@list.ru

**Keywords:** *Thinopyrum elongatum*, plant antimicrobial peptides, defensin, *γ*-core, plant pathogens, human pathogens, novel antimicrobials

## Abstract

Fungal and bacterial pathogens significantly impact global crop yields, causing substantial economic losses and food insecurity. While chemical pesticides are effective, their excessive and improper use poses risks to the environment and human health. Antimicrobial peptides (AMPs)—components of innate immunity in plants and animals—are promising candidates for the development of novel, eco-friendly antimicrobials for agriculture and medicine. This study explores the antimicrobial activity of several *γ*-core peptides derived from defensins of *Thinopyrum elongatum*, a wild plant species known for its stress resistance. All peptides carried a net positive charge. 3D structural modeling indicated that most peptides adopted an *α*-helical conformation, with one predicted to form an anti-parallel *β*-hairpin structure. The conservation of the *γ*-core peptide sequences across Poaceae defensins was demonstrated, underscoring the importance of these peptide regions in biological functions of defensins. Antimicrobial assays demonstrated that all peptides exhibited broad-spectrum activity, with efficacy depending on the peptide’s amino acid sequence, 3D structure, and the pathogen tested. Notably, the peptide with the highest positive charge and *β*-hairpin structure showed the strongest pathogen inhibition. Additionally, synergistic interactions between some peptides against *Fusarium oxysporum*, which enhanced their antimicrobial effects, were shown. Our findings highlight the potential of wheatgrass *γ*-core peptides as templates for developing new peptide-based antimicrobials for agricultural and medical applications.

## 1. Introduction

Antimicrobial peptides (AMPs) form a crucial part of innate immunity in both animals and plants. Despite their diverse structures and functions, AMPs share common characteristics, such as a low molecular weight (less than 10 kDa) and typically a net positive charge, which facilitates their interaction with negatively charged components of pathogen membranes. Up to 50% of an AMP’s sequence usually consists of hydrophobic residues, enabling van der Waals interactions with membrane lipids [1]. Plant AMPs are commonly cysteine-rich peptides containing from 4 to 16 cysteine residues that form disulfide bonds, stabilizing their three-dimensional structure. AMPs employ various mechanisms to inhibit the growth of pathogenic microbes, with their mode of action depending on their physicochemical properties, such as primary and secondary structure, length of the molecule, net charge, and amphiphilicity. In addition to disrupting membrane integrity, some AMPs target intracellular processes within pathogens. AMPs from various plant species differ in their antimicrobial potency, spectrum of activity, and modes of action against different groups of pathogens. Many are effective against both plant and human pathogens, including strains exhibiting multiple drug resistance [2], and are non-toxic to mammalian cells [3].

The distinctive antimicrobial properties of AMPs have captured the interest of researchers, prompting them to explore these peptides as potential alternatives to traditional antibiotics and antifungals for combating infectious diseases in plants, humans, and animals. AMPs offer several advantages over conventional antibiotics, one of the most notable being their rapid action. Studies have demonstrated that, at concentrations exceeding the threshold, AMPs tend to act more quickly than antibiotics [4,5,6,7,8]. AMPs are effective against a broad spectrum of pathogens—including bacteria, fungi, viruses, and parasites [5]—while antibiotics typically target only bacteria, and fungicides only fungi. An important advantage of AMPs is their ability to kill pathogens at all stages of development, including non-dividing cells and spores [9,10]. Additionally, AMPs can act on biofilms—complex microbial communities responsible for many infectious diseases and often resistant to antibiotics [11,12]. Another important characteristic of AMPs is their low propensity for resistance development in pathogens, which is significantly lower than that observed with antibiotics [5,13,14,15]. Beyond their direct antimicrobial activity, AMPs also possess immunomodulatory activity [16,17]. A notable feature of AMPs is their capacity for synergistic action, where different peptides work together with each other, with other components of the plant immune system, and in combination with fungicides and antibiotics [18,19,20,21,22]. A key advantage of AMPs as potential new antimicrobial agents is their relative ease of synthesis, as they are short chains of amino acids [23]. They are also environmentally safe because they naturally decompose without forming toxic products and can be used as safe antibiotics that do not harm beneficial microbial communities [24].

All of these features make AMPs highly promising molecules for the development of new antimicrobials in agriculture and medicine. Using AMPs for plant protection can help reduce the reliance on traditional antibiotics and fungicides, thereby benefiting the environment. Moreover, the combined use of AMPs and chemical plant protection agents can slow down the development of resistance to these chemicals and extend their effectiveness [25].

Despite obvious advantages, the application of AMPs in medicine and agriculture faces certain challenges associated with their sensitivity to proteolytic degradation, resulting in a short lifetime accompanied by rapid removal from the circulation, poor oral bioavailability, possible cytotoxicity and immunogenicity [26]. In order for AMPs to be used as plant protection agents, they must maintain stability and withstand adverse environmental factors such as extreme temperatures, UV radiation, pH changes and others. Some of these limitations can be overcome by modifying the peptide structure; for example, to increase resistance to proteases, D-amino acids can be incorporated into the peptide structure, or they can be encapsulated for protection against unfavorable factors. However, all these measures complicate the process and increase the cost of the final product.

In agriculture, it is also necessary to assess the impact of AMPs on non-target organisms. Natural plant AMPs are predominantly non-toxic, which is not the case for synthetic peptides. The toxicity of the peptide, as well as its stability, can be reduced by altering the peptide structure while maintaining activity. The question of how to apply the peptide to plants remains open. Spraying requires large quantities of peptides (kg/ha). Endotherapy is only applicable to woody crops and is also underdeveloped from a technical standpoint. It seems reasonable to include AMPs in formulations where they act synergistically with other components.

Finally, a key factor that remains to be addressed for the implementation of AMPs in agricultural practice is cost reduction in the industrial-scale production of AMPs. AMPs can be produced by several methods. They can be extracted from plant tissues, chemically synthesized, or obtained through heterologous expression in various organisms [27]. In plants, the content of AMPs is usually very low, and the process of peptide purification is labor-intensive and time-consuming. Modern methods of AMP production include liquid-phase and solid-phase synthesis, as well as heterologous expression. The most widespread systems for expression are in bacteria and yeasts. The bacterial species *Escherichia coli* is most commonly used for expression due to its high growth rate and well-developed recombinant methods. Although many AMPs are expressed in bacteria, there are several limitations associated with the toxic effects of AMPs on host cells, the lack of post-translational modifications in the recombinant peptide, and the need for carrier (fusion) proteins, which must be addressed to achieve efficient production. For AMP expression in yeasts, *Pichia pastoris* and *Saccharomyces cerevisiae* are typically used. In recent decades, plant systems have increasingly been employed for AMP production. Tobacco is one of the most well-studied platforms for the expression of recombinant proteins. Defense peptides can be obtained either by creating transgenic plants or through transient expression, both with sufficiently high yields.

Solid-phase chemical synthesis remains the major AMP production strategy. However, it is a relatively expensive method, especially for long peptides. One gram of a decapeptide costs several hundred dollars [27]. Nevertheless, ongoing research aimed at improving AMP production technologies offers hope that unresolved issues will be successfully addressed.

Since the main limiting factor for the practical application of AMPs as plant protection agents is the high cost associated with large-scale peptide synthesis, the research into minimal functional domains within AMPs is of great importance. Recent studies have identified the *γ*-core region, a sequence of 10–15 amino acids containing the motif GXCXnC, as a functionally significant region of AMP molecules, which largely determines antimicrobial activity [28]. Short peptides derived from the *γ*-core regions are especially promising for the development of next-generation antimicrobials.

*Thinopyrum elongatum* (Host) D.R. Dewey is a perennial, cross-pollinated grass belonging to the Poaceae family that grows naturally in various environments and serves as an excellent forage crop. It exhibits high resistance to both biotic and abiotic stresses, including fungal diseases, low temperatures, soil salinity, and drought. Additionally, this species is known for its high yield potential [29]. Due to higher protein content in grain compared to wheat, *T. elongatum* has been considered as a potential donor for enhancing the technological and nutritional qualities of soft wheat varieties. The resilience of wheatgrass to environmental challenges makes it a valuable subject for studying mechanisms of resistance to pathogens and abiotic stress factors. Several resistance genes to fungal diseases conferring protection against specific pathogen races have been discovered in its genome, which made *T. elongatum* a unique donor of resistance traits in wheat breeding programs [29]. Recently, we have identified the repertoire of AMP genes responsible for providing broad-spectrum resistance in *T. elongatum* genome by in silico mining [30].

In this study, we continue to explore the antimicrobial potential of *γ*-cores derived from plant AMPs, and this time, we focus on the *γ*-cores of *T. elongatum* defensins. We synthesized a number of wheatgrass defensins’ *γ*-cores and studied their antimicrobial activity against a panel of plant pathogens and human opportunistic fungi. We discovered the peptides with high antimicrobial activity that could find application as novel plant crop protection agents and innovative antifungals in medicine.

## 2. Results

### 2.1. Design of γ-Core Peptides

**Figure 1:**
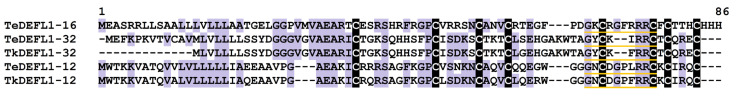
Multiple sequence alignment of *Thinopyrum elongatum* and *Triticum kiharae* DEFLs. The sequences of *γ*-cores are underlined. Cysteine residues are shaded black, and identical amino acids are shaded lavender.

Three wheatgrass defensins, TeDEFL1-12, TeDEFL1-16 and TeDEFL1-32, discovered in *T. elongatum* genome [30], were selected for the synthesis of the *γ*-core motif peptides (Figure 1). The peptides were designed as follows: they contained the sequence of the *γ*-core with GXCXnC motif and additionally six amino acids at the C-terminus including two cysteine residues (Table 1). The *γ*-core containing peptides were produced by chemical synthesis and purified by high-performance liquid chromatography. Two parent wheatgrass defensins TeDEFL1-12 and TeDEFL1-32 are highly homologous to the wheat DEFLs (TkDEFL1-12 and TkDEFL1-32, respectively) identified in *Triticum kiharae* transcriptomic data [31], with their *γ*-cores differing by the single substitutions L7F and I5F, respectively. Our previous work showed that the peptides containing these *γ*-cores, DEFL1-12_62–77_ and DEFL1-32_55–68_, possessed antimicrobial activity [32]. To reveal the role of these substitutions in the biological activity of *γ*-core peptides, these two wheat peptides were also synthesized for comparison.

### 2.2. Homologs of Synthetic γ-Core Peptides

**Figure 2:**
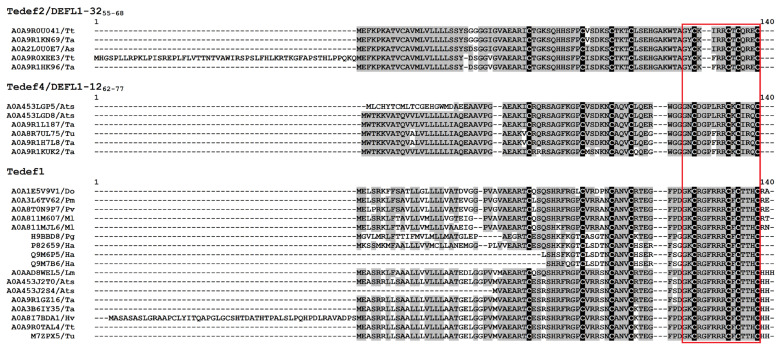
Multiple sequence alignment of defensin-like peptides containing the synthetic *γ*-core peptide sequences of wheatgrass and wheat. The sequences of the synthetic peptides are framed. Cysteine residues are shaded black, and identical amino acids are shaded grey. The following abbreviations were used: Tt, *Triticum turgidum*; Ta, *T. aestivum*; Tu, *T. urartu*; As, *Avena sativa*; Ats, *Aegilops tauschii* subsp. *strangulata*; Do, *Dichanthelium oligosanthes*; Pm, *Panicum miliaceum*; Pv, *P. virgatum*; Pg, *Panax ginseng*; Ml, *Miscanthus lutarioriparius*; Ha, *Helianthus annuus*; Lm, *Lolium multiflorum*; and Hv, *H. vulgare*.

It was of interest to determine how broadly the selected peptide sequences are distributed within the plant kingdom, and so, a Peptide search was conducted against the UniProt database [33], with the sequences of the synthesized peptides as queries. As a result, 28 plant defensin sequences were identified (Figure 2).

The highest number of defensin-like peptides (17) contained the *γ*-core sequence Tedef1. Of them, thirteen defensins were from plants of Poaceae family (*Triticum aestivum*—2, *T. urartu*—1, *T. turgidum*—1, *Hordeum vulgare*—1, *Panicum miliaceum*—1, *P. virgatum*—1, *Aegilops tauschii* subsp. *strangulata*—2, *Lolium multiflorum*—1, *Miscanthus lutarioriparius*—2, *Dichanthelium oligosanthes*—1). Three defensins belonged to *Helianthus annuus* (Asteraceae family) and one defensin—to *Panax ginseng* (Araliaceae family). The Tedef2 sequence was found in three defensins from *T. aestivum*, *T. turgidum*, and *Avena sativa*, while DEFL1-32_55–68_ was discovered in two defensins from *T. aestivum* and *T. turgidum*. The Tedef4 sequence was identified in two defensins from *A. tauschii* subsp. *strangulata* and one defensin from *T. aestivum*. Two DEFL1-12_62–77_ containing defensins were found in *T. aestivum* and one in *T. urartu*. Accordingly, the sequences of synthetic peptides occur more frequently in plant defensins of the Poaceae family. *T. aestivum* contained the greatest number of such sequences (7), with all five synthetic peptides being identified. The wide distribution of synthetic peptide sequences among the defensins of Poaceae plants indicates functional significance of this “extended” *γ*-core region of the molecule in the defensins’ functions.

### 2.3. Physicochemical Characteristics of the Synthetic Peptides

The peptides contain from 14 to 16 amino acid residues (Table 1). Their molecular weights range from 1719.05 to 1931.30 Da. All peptides are cationic (Table 2). The pI (isoelectric point) values vary from 8.94 in Tedef2 and DEFL1-32_55–68_ to 9.89 in Tedef1. The net charge is in the range from +3 to +5 (Table 2). The positive charge has been acknowledged to be crucial for the initial electrostatic interaction of the peptide with the negatively charged cell membranes of pathogenic microorganisms [34]. The highest positive charge of +5 observed in Tedef1 results from the presence of five positively charged residues (Arg or Lys). All other peptides contain three positively charged residues, leading to a net charge of +3. The aliphatic index, which correlates positively with thermal stability, varies from 0 to 48.75, being the highest in Tedef4 (Table 2). Both Tedef1 and DEFL1-32_55-68_ have an aliphatic index of 0. The Boman index, which indicates protein-binding potential, ranges from 3.39 in Tedef4 to 4.02 in DEFL1-32_55–68_. These values suggest that these peptides can interact with a broad spectrum of proteins. The ratio of hydrophobic amino acid residues is 36% in Tedef2 and DEFL1-32_55–68_ and 38% in Tedef1, Tedef4, and DEFL1-12_62–77_. The hydrophobic moments (μH), indicating the amphipathicity of an *α*-helix, were calculated. The highest hydrophobic moment of 0.318 was in Tedef4. The GRAVY (Grand Average of Hydropathy) index is negative for all peptides (Table 2), suggesting that they are predominantly hydrophilic and dissolve in water. All peptides were predicted to possess antimicrobial activity.

### 2.4. Three-Dimensional Structure Modeling of Synthetic Peptides

**Figure 3:**
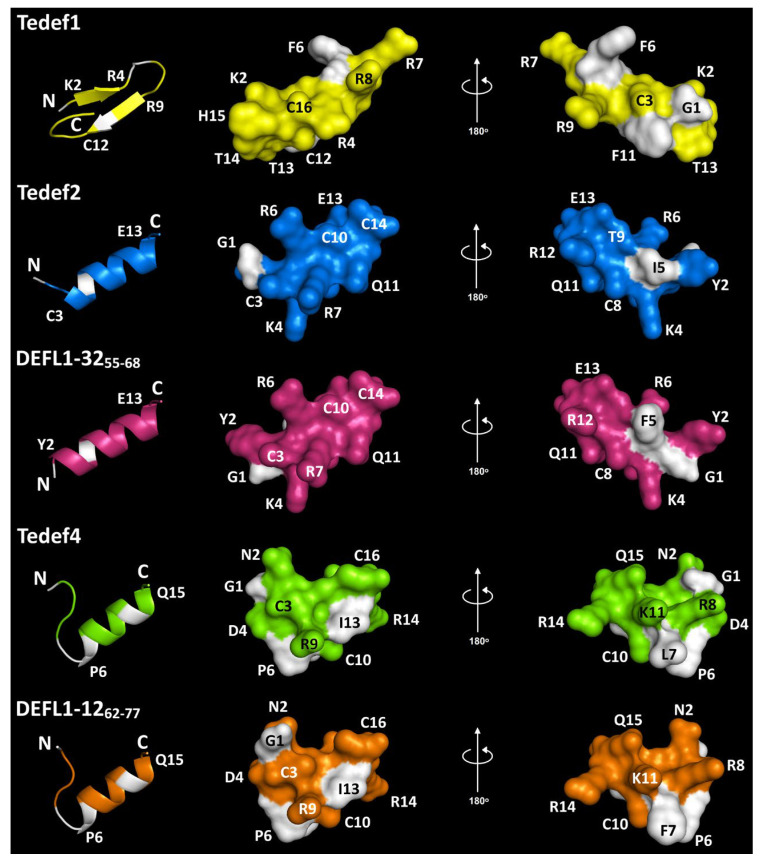
3D molecular modeling of *T. elongatum* and *T. kiharae γ*-core-containing synthetic peptides: spatial (ribbon representation) and surface structures. N- and C-termini are marked with N and C, respectively. Non-polar residues are shown in white, polar residues are colored. Modeling was carried out using PEP-FOLD4 [35].

The 3D structure of synthetic peptides was predicted with PEP-FOLD4 (Figure 3) [35]. Tedef1 was predicted to adopt anti-parallel *β*-hairpin conformation, with *β*-sheets located from K2 to R4 and from R9 to C12. Four other peptides contained an *α*-helical region of different length (10–12 amino acid residues) and unstructured N- and C-terminal “tails” (Figure 3).

In Tedef2 and DEFL1-32_55–68_, the *α*-helical region was predicted to occupy most of the peptide’s molecule, with the longest *α*-helix of 12 residues in DEFL1-32_55–68_ (Figure 3). The substitution I5F led to the extension of the *α*-helix and a shift in positions of G1 and Y2 residues, resulting in the formation of a single hydrophobic cluster composed of G1 and F5 in DEFL1-32_55–68_ (Figure 3). Tedef4 and DEFL1-12_62–77_ have shorter *α*-helices of 10 residues in length each. The spatial structures of these two peptides are very similar. However, the only substitution L7F resulted in a significant change in the position of G1, and moreover, the Phe side chain is much bulkier than that of Leu. Most peptides exhibit amphipathic properties, with hydrophobic and polar residues positioned on the opposite sides of the molecule. Furthermore, positively charged residues tend to cluster together: R4, R7, R8, and R9 in Tedef1; K4, R6, and R7 in Tedef2 and DEFL1-32_55–68_; and R8, K11, and R14 in Tedef4 and DEFL1-12_62–77_.

### 2.5. Antimicrobial Activity of Synthetic Peptides

The antimicrobial activity of synthetic *γ*-core peptides was assessed against the pathogenic fungi (*Fusarium oxysporum*, *F. culmorum*, *F. solani*, *F. verticillioides*, *Aspergillus unguis*, *Bipolaris sorokiniana*, *Botrytis cinerea*, *Rhizoctonia solani*, *Penicillium gladioli*, *Candida albicans*, *C. tropicalis*, and *Cryptococcus neoformans*) and bacteria (*Clavibacter michiganensis*, *Pseudomonas savastanoi*, and *Pectobacterium carotovorum*).

Studies of the antifungal activity of five synthetic peptides showed that each peptide inhibited the growth of filamentous fungi, with the level of inhibition varying depending on the peptide tested (Table 3). Tedef1 was active against all tested fungi, with IC_50_ ranging from 12.5 to 107.6 μM, thus demonstrating the broadest activity spectrum. Tedef1 exhibited strong antifungal activity against *F. culmorum* (IC_50_ = 12.5 μM), *F. verticillioides* (IC_50_ = 16.9 μM), and *F. solani* (IC_50_ = 26.8 μM), less effectively this. This peptide inhibited the growth of *F. oxysporum* and *A. unguis* (IC_50_ = 34.3 and 40.3 μM, respectively) less effectively. It is worth noting, that Tedef1 was the most efficient against *Fusarium* species among all the peptides. Moreover, this peptide was also the most active against *P. gladioli*, *B. sorokiniana*, *B. cinerea*, and *R. solani*, with the latter two pathogens being insensitive to the other peptides at the tested concentrations (Table 3). Of the two peptides, Tedef2 and DEFL1-32_55–68_, which differ by one substitution I5F, peptide Tedef2 (I5) was more effective against *F. culmorum*, *F. oxysporum*, *P. gladioli*, and *B. sorokiniana*, but less active against *A. unguis*. This substitution had almost no effect on the inhibition of *F. verticillioides*; the inhibitory activities of these two peptides were similar (IC_50_ = 73.5 and 79.5 μM, respectively). Additionally, DEFL1-32_55–68_ showed weak growth inhibition of *F. solani* (Table 3). The Tedef4/DEFL1-12_62–77_ peptide pair with L7F substitution inhibited the growth of the same spectrum of fungi, but with different efficiencies. Both showed the best activity against *A. unguis* (IC_50_ = 2.1 and 2.0 μM, respectively) and were the most effective growth inhibitors of this pathogen. The activity against other pathogens was much weaker, with DEFL1-12_62–77_ being more effective against *F. culmorum* and *P. gladioli*, and Tedef4 (L7) —against *F. verticillioides* and *B. sorokiniana*. Both showed no inhibitory effect on *F. oxysporum*, *F. solani*, *B. cinerea*, and *R. solani* within the tested concentration range (Table 3).

In contrast to multicellular fungi, synthetic *γ*-core peptides inhibited the growth of yeasts more effectively. Tedef1 was the most potent inhibitor of yeasts with an IC_50_ of 5.9 μM against *C. tropicalis*, 7.3 μM against *C. albicans*, and 10.6 μM against *C. neoformans* (Table 3). All other peptides were ineffective against *C. albicans* at the tested concentrations. DEFL1-32_55–68_ was slightly less active than Tedef1 with IC_50_ values of 7.8 μM and 12.5 μM against *C. tropicalis* and *C. neoformans*, respectively (Table 3). Tedef2 was the most efficient against *C. neoformans,* with the lowest IC_50_ value of 9.9 μM. *C. tropicalis* was less sensitive to this peptide, with an IC_50_ of 26.5 μM. Tedef4 and DEFL1-12_62–77_ exhibited similar levels of antimicrobial activity against yeasts: their IC_50_ values were 29.8 and 28.6 μM against *C. neoformans* and 100.2 and 96.0 μM against *C. tropicalis*, respectively (Table 3).

The *γ*-core peptides were able to suppress the growth of two bacteria, the Gram-positive *C. michiganensis* and the Gram-negative *P. savastanoi*, but failed to suppress the growth of another Gram-negative bacterium, *P. carotovorum,* within the tested concentration range. Tedef1 was the most efficient in inhibiting bacterial growth with IC_50_ of 29.4 and 45.2 μM against *C. michiganensis* and *P. savastanoi*, respectively. Tedef2 was less active against these pathogens (Table 3). Moreover, substitution of I5F in DEFL1-32_55–68_ resulted in a decrease in activity against *P. savastanoi* and total loss of activity against *C. michiganensis*. A similar effect was observed for a pair of peptides, Tedef4 and DEFL1-12_62–77_, and the L7F substitution resulted in the total loss of inhibitory activity against *C. michiganensis* in DEFL1-12_62-77_. Both peptides were inactive against *P. savastanoi* at the concentrations tested (Table 3).

### 2.6. Synergistic Interactions of the γ-Core Peptides

**Figure 4:**
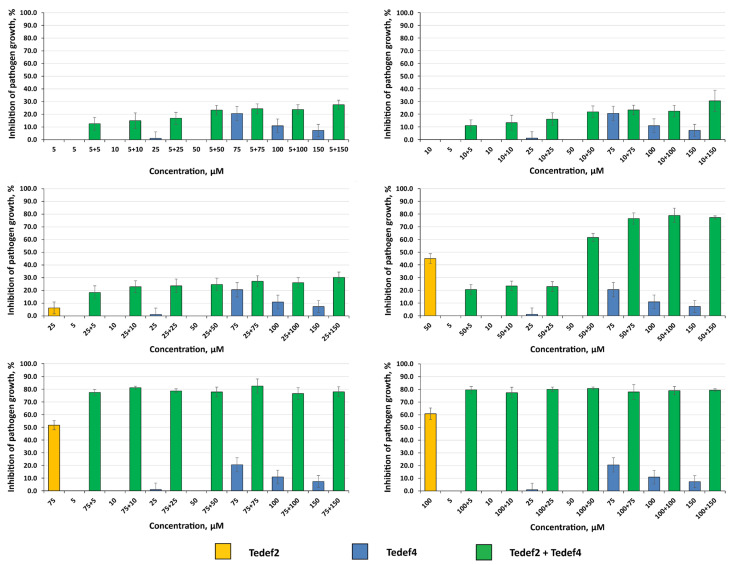
Inhibition of *F. oxysporum* growth by peptides Tedef2 and Tedef4 co-applied in various combinations of concentrations. The first figure in the combinations refers to Tedef2, and the second to Tedef4. Results are expressed as the mean of three experiments, and SDs are shown.

To reveal the possible synergism of action of the *γ*-core peptides, peptides Tedef2 and Tedef4 derived from *T. elongatum* defensins were tested against two different pathogens—the fungal pathogen *F. oxysporum* and the bacterium *C. michiganensis*. The results of synergism evaluation against *F. oxysporum* are shown in Figure 4.

The activity of the peptides against *F. oxysporum* tested individually varied significantly, Tedef2 being much more active than Tedef4 within the tested concentration range (Figure 4). Co-application of these peptides resulted in an enhanced antifungal effect compared to the action of the peptides taken alone (Figure 4, Table 4). Moreover, in almost all cases, except when the concentrations of Tedef2 and Tedef4 peptides were 50 μM and 5–25 μM, respectively, the observed levels of inhibition in co-applications (Er) exceeded the calculated additive effects of the peptides (Ee) (Table 4). However, these differences were significant (t-test for independent variables, *p* ≤ 0.05) only for certain combinations of peptide concentrations—5 or 10 µM Tedef2 and 50 µM Tedef4; 50 µM Tedef2 and 150 µM Tedef4; and 75 µM Tedef2 and 5–50 µM Tedef4 (Table 4)—which means that these combinations of peptide concentrations resulted in a synergistic interaction, while at other combinations, the enhancement of antifungal activity was due to the additive action of peptides.

**Figure 5:**
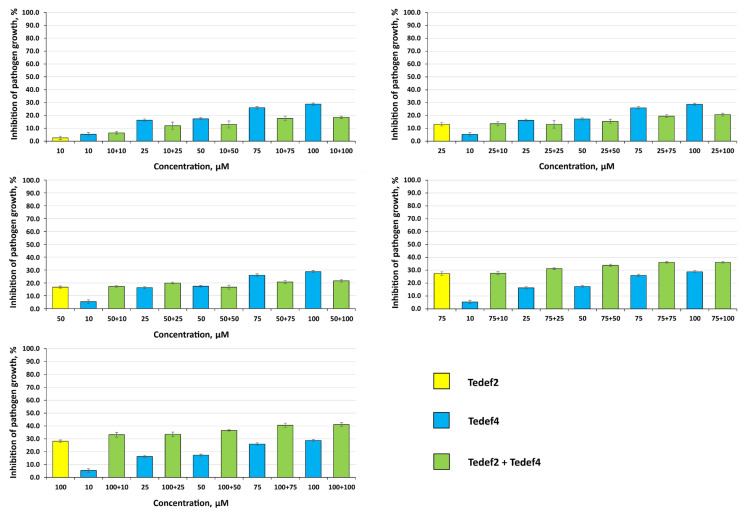
Inhibition of *C. michiganensis* growth by peptides Tedef2 and Tedef4 co-applied in various combinations of concentrations. The first figure in the combinations refers to Tedef2, and the second to Tedef4. Results are expressed as the mean of three experiments, and SDs are shown.

The activity of the peptides Tedef2 and Tedef4 against *C. michiganensis* was approximately the same, as both peptides exhibited low inhibitory activity when tested individually (Figure 5). At the same time, in most cases, observed levels of inhibition in co-applications exceeded the effects of the peptides alone (Table 5), although these levels remained rather low.

However, the Er values only exceeded the calculated Ee values at one combination of peptide concentrations (100 µM Tedef2 and 10 µM Tedef4), and this difference was not statistically significant (Table 5). Therefore, no synergistic effect against *C. michiganensis* was detected. The absence of synergistic interactions between the tested peptides against *C. michiganensis* may indicate differences in the peptides’ mode of action against filamentous fungi, or, more specifically, *Fusarium* fungi, and Gram-negative bacteria, or, more specifically, *C. michiganensis*.

### 2.7. Staining with Propidium Iodide

**Figure 6:**
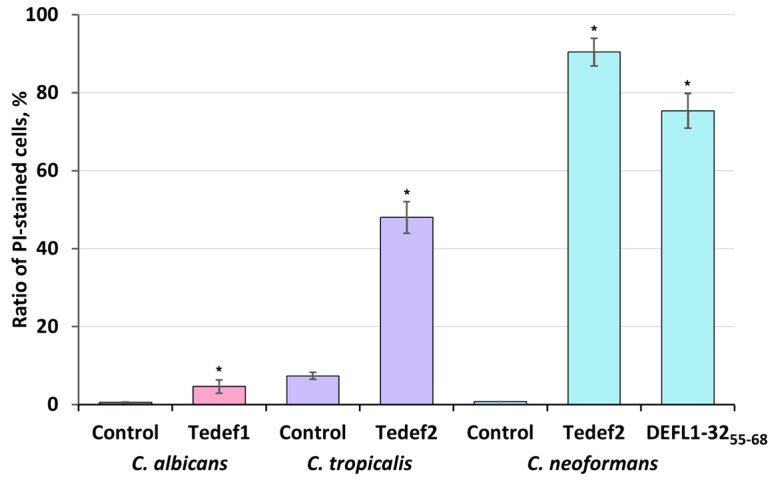
Effect of the *γ*-core motif peptides at a concentration of 100 µM on penetration of propidium iodide (PI) into pathogen cells. Untreated cells were used as a negative control. Bars represent mean ± SD. Asterisks indicate significant differences between treated and control cells (Student’s *t*-test, *p* ≤ 0.05).

The ability of the *γ*-core peptides to compromise the integrity of pathogen cytoplasmic membranes as one of the possible mechanisms of action was assessed by measuring the percentage of cells stained with propidium iodide (PI) after treatment with the peptides. Three peptides, Tedef1, Tedef2, and DEFL1-32_55-68_, and three pathogens, *C. albicans*, *C. tropicalis*, and *C. neoformans*, were used for the staining experiment. The results were quantified by flow cytometry (Figure 6).

All the peptides tested caused the accumulation of the fluorescent dye within the cells of three yeast species, indicating membrane permeabilization as one of the mechanisms of the peptides’ action (Figure 6).

## 3. Discussion

Pathogenic microorganisms are the cause of diseases in both plants and humans. Among these, fungi pose the greatest biotic threat to crop production [36]. The primary fungal pathogens affecting plants include *Fusarium* spp., *Alternaria* spp., *Pythium* spp., *Colletotrichum* spp., *Puccinia* spp., etc. [37,38]. Fungal diseases impact all major crops that are vital for global nutrition, including rice, wheat, maize, soybean, and potato [39]. Although yield losses vary, the overall disease burden is estimated to reduce harvests by 10 to 23% [39,40]. Additionally, post-harvest losses from fungal infections account for another 10–20%, and in developing countries, these losses can reach as high as 50% [41]. Fungal infections in crops also lead to the production of mycotoxins—compounds produced by fungi that are highly toxic to humans and livestock [42]. It is estimated that about 25% of all crops contain “unsafe” levels of mycotoxins for human consumption, and 60–80% of crops have detectable levels of these toxins [42]. Hundreds of different mycotoxins have been identified, including aflatoxins produced by *Aspergillus* species, which contaminate cereals, oilseeds, spices, and nuts [43]. Mycotoxins from *Fusarium* species—such as trichothecenes, fumonisins, and zearalenone—also significantly contribute to crop losses both in the field and during storage [44,45].

Fungicides continue to be the primary tool in agriculture for managing plant diseases [46]. The advantages of pesticides in boosting crop yields are well recognized: they help protect plants from pests and diseases, leading to increased production and improved quality. Without the use of pesticides, global food production would be significantly affected, resulting in greater food insecurity and higher prices for consumers. However, the widespread use of pesticides also has its limitations and drawbacks. Among the six main classes of agricultural fungicides, only three—azoles, strobilurins, and succinate dehydrogenase inhibitors (SDHIs)—are predominant in the market. As a result, resistance to these three classes has developed in major crop pathogens [47]. Additionally, research indicates that only about 1% of total pesticide applications actually reach the intended targets. The majority of these chemicals impact non-target organisms and ecosystems, leading to soil degradation, water and air pollution, food contamination, and health problems in animals and humans [46].

Beyond their negative effects on crop yield and quality, many fungi also cause fungal infections, or mycoses, in humans. Globally, fungi are believed to be responsible for over one billion superficial infections each year, including conditions such as nail and hair infections, vaginal yeast infections, and oral thrush [48,49]. While fungal diseases are generally not life-threatening for healthy individuals, they often require multiple treatments, which can lead to the development of antifungal resistance. Invasive fungal infections, which typically occur in people with weakened immune systems, are less common but are associated with approximately 2.5 million deaths annually [50]. *Candida* species are the most prevalent opportunistic fungi infecting humans, which are capable of causing severe bloodstream infections (fungemia) with mortality rates reaching up to 75%, as well as superficial infections [48]. *Cryptococcus neoformans* is another widespread pathogen, particularly affecting immunocompromised populations [51]. Cryptococcal meningitis is one of the most serious HIV-related opportunistic infections, especially in developing countries. For instance, in 2009, in sub-Saharan Africa alone, cryptococcal meningitis accounted for around 17% of AIDS-related deaths [52]. Certain fungi, such as *Fusarium* species, can infect both plants and animals, causing diseases across different hosts [53].

Treatment of fungal infections in humans has primarily relied on four classes of systemic antifungal drugs: triazoles, echinocandins, polyenes, and the pyrimidine analogue 5-flucytosine (5-FC) [54,55]. However, these drugs are limited in chemical diversity, often have host toxicity, possess a narrow activity spectrum, and can promote the development of resistance in pathogens.

In addition to pathogenic fungi, bacteria that cause diseases in plants also lead to substantial yearly losses in crop production worldwide [56]. The main bacterial genera responsible for plant diseases include *Xanthomonas*, *Agrobacterium*, *Pseudomonas*, *Xylella*, *Ralstonia*, *Erwinia*, and *Clavibacter* [57]. It is estimated that bacterial diseases contribute to approximately 10–13% of total global losses in crop yields [58]. The use of antibiotics to control bacterial infections faces similar limitations as fungicides do in managing fungal diseases, both in agriculture and medicine [59].

From this overview, it is clear that there is an urgent need for the development of new antimicrobial agents.

In this study, we assessed the antimicrobial activity of three wheatgrass *γ*-core peptides against a broad panel of phytopathogens and opportunistic fungi affecting humans. Additionally, two wheat *γ*-core peptides with high sequence similarity to the wheatgrass peptides were examined for comparison purposes. The peptides Tedef2 and DEFL1-32_55–68_ differ by a single amino acid substitution I5F, while Tedef4 and DEFL1-12_62–77_ by a single substitution L7F (Table 1). Both substitutions involve replacing a hydrophobic amino acid with another hydrophobic one, resulting in identical net charges at pH 7, pI values, hydrophobic residue ratios, and μH values within each pair (Table 2). Structural modeling of these peptide pairs revealed that they all adopt an *α*-helical conformation. Although their overall structures are similar, some differences were observed (Figure 3). The I5F substitution caused an extension of the *α*-helix and the formation of a single hydrophobic cluster involving G1 and F5 in the wheat DEFL1-32_55–68_. In the pair Tedef4 and DEFL1-12_62–77_, the L7F substitution led to notable surface differences in the peptides (Figure 3). The amino acid sequence and 3D structure of Tedef1 differ significantly from those of the other four peptides. Unlike the previously mentioned pairs, Tedef1 adopts an anti-parallel *β*-hairpin structure and exhibits distinct physicochemical properties, being more basic with a net charge of +5 and a pI of 9.89 (Table 2, Figure 3).

Analysis of the antimicrobial properties of five *γ*-core peptides revealed that all exhibited broad-spectrum activity, effectively targeting multiple pathogens. Among them, Tedef1 with the sequence RGFRRR in the *γ*-core region demonstrated the strongest activity against most tested multicellular fungi (excluding *A. unguis*), yeasts, and bacteria, likely due to its high net charge and *β*-hairpin structure. However, these advantageous features did not seem to benefit its activity against *A. unguis*. The peptide pair Tedef4/DEFL1-12_62–77_, featuring an L7F substitution, showed the most effective activity against this mold fungus. It is noteworthy that the RGFRRR sequence within the *γ*-core region is widely distributed in defensins across a diverse range of plant families and species [60]. In this research, we show that in plants of the Poaceae family, the entire defensin sequence containing RGFRRR in the *γ*-core is highly conserved, emphasizing the important role of these peptide molecules in plant defense and potentially other cellular processes (Figure 2).

Within the pair Tedef2/DEFL1-32_55–68_, the peptide Tedef2 (with I5) was generally more active than DEFL1-32_55–68_ (with F5) against most multicellular fungi; the latter was only more effective against the fungus *A. unguis*. The I5F substitution did not influence their activity against *F. verticillioides*. Overall, the Tedef4/DEFL1-12_62–77_ pair, with the L7F substitution, exhibited weaker activity against most multicellular fungi compared to Tedef2/DEFL1-32_55-68_, except for its notably strong activity against *A. unguis*. The role of the L7F substitution varied depending on the fungal species: Tedef4 was more effective against *F. verticillioides* and *B. sorokiniana*, whereas DEFL1-12_62–77_ was more potent against *F. culmorum* and *P. gladioli*.

Regarding yeast inhibition, the I5F substitution in the Tedef2/DEFL1-32_55–68_ pair had differential effects based on the target pathogen. Tedef2 (I5) was more active against *C. neoformans*, while DEFL1-32_55–68_ (F5) showed better activity against *C. tropicalis*. In the Tedef4/DEFL1-12_62–77_ pair, activity against these yeasts was nearly identical, suggesting that the L7F substitution does not significantly influence antifungal activity against these species.

Overall, when examining the effect of amino acid substitutions on antifungal activity: for the Tedef2/DEFL1-32_55–68_ pair, Ile5 was beneficial for inhibiting almost all fungi except *A. unguis* and *C. tropicalis*; for the Tedef4/DEFL1-12_62–77_ pair, the influence was ambiguous—Leu favored some fungi, while Phe was better against others, and against yeasts, the residue at position 7 (L or F) had no significant effect on the activity.

Regarding antibacterial activity, it was generally weaker across the peptides. Nonetheless, Tedef2 was more effective than DEFL1-32_55–68_ against *C. michiganensis* and *P. savastanoi*, and Tedef4 outperformed DEFL1-12_62–77_ against *C. michiganensis*. This indicates that the bulky Phe residue may impair the antibacterial efficacy of DEFL1-32_55–68_ and DEFL1-12_62–77_.

Thus, these results indicate that even minor, single amino acid changes in the defensin-derived *γ*-core peptides can markedly affect their ability to combat microbes. Furthermore, the *γ*-core peptides from wheatgrass generally exhibited greater activity against plant pathogens than those from wheat. This may partly account for the increased resistance to diseases observed in the wild species *T. elongatum* compared to cultivated wheat. Moreover, the Tedef2/DEFL1-32_55–68_ pair was more active than the Tedef4/DEFL1-12_62–77_ pair, despite having identical molecular charge. It can be assumed that this may be related to the spatial structure of the peptides: the *α*-helix occupies almost the entire length of the Tedef2/DEFL1-32_55–68_ peptides, resulting in a more compact and stable structure (Figure 3).

In our research, we demonstrated that some wheatgrass *γ*-core peptides induce membrane permeabilization in yeast cells, representing one of their mechanisms of antimicrobial action. This aligns well with previous findings from our studies on the *γ*-core peptides derived from wheat, tomato, and *Filipendula ulmaria* AMPs [32,61,62]. Similar effects have also been observed by other researchers working with filamentous fungi. For example, the *γ*-core peptide SmAP*γ*_27–44_ from *Silybum marianum* defensin DefSm2-D was shown to disrupt the plasma membrane of *Fusarium graminearum*, leading to cytoplasmic disorganization [63]. Using a liposome model, it was further demonstrated that this peptide can penetrate cells by inserting a tryptophan residue into the membrane’s hydrophobic moiety [63]. The *γ*-core peptide from MtDef4 was found to permeabilize *B. cinerea* membranes and then internalize into the vacuole, with the sequence motif RRRW identified as critical for its antifungal activity [64]. Additionally, a synthetic peptide based on the *γ*-core motif of PvD1 defensin caused rapid acidification and permeabilization of *Candida buinensis* cells, resulting in cell death through apoptosis, along with mitochondrial dysfunction, oxidative burst, and activation of metacaspases [65]. A modified *γ*-core peptide derived from the olive defensin OefDef1.1 was shown to rapidly disrupt the plasma membrane of *B. cinerea* and accumulate in the vacuole, causing vacuolar expansion and cell death. It also inhibited protein synthesis, suggesting a role as a translation inhibitor. Mutational analysis identified the ^7^RHSKH^11^ motif as essential for its antifungal activity, and transcriptomic studies revealed upregulation of genes involved in membrane transport, lipid and carbohydrate metabolism, oxidative stress response, and proteolysis, with downregulation of mitochondrial genes [66]. Proteomic analysis of *Escherichia coli* exposed to a truncated *γ*-core defensin from *Amaranthus tricolor* showed that its antibacterial effect involves membrane disruption and metal sequestration, limiting enzyme cofactor availability [67]. Interestingly, recent research suggests that *γ*-core peptides can inhibit plasma membrane H^+^-ATPases at low concentrations without permeabilizing the membrane, potentially disrupting ionic balance and leading to cell death [68]. Whether wheatgrass peptides share this mechanism remains to be investigated.

In this study, we demonstrated that two wheatgrass *γ*-core peptides work together synergistically to inhibit *F. oxysporum*. This observation aligns with existing research showing that different AMPs can interact synergistically, as well as combinations of AMPs with antibiotics. For instance, synergistic effects have been reported for interactions between *Raphanus sativus* and *Brassica napus* 2S albumins, wheat and barley thionins, and combinations of thionins with Bowman-Birk inhibitors against filamentous fungi and Gram-positive bacteria [69]. Our previous work also confirmed synergistic effects involving *γ*-core peptides from meadowsweet AMPs [62]. Radish defensins RsAFP1 and RsAFP2 have been shown to work synergistically with caspofungin to combat *Candida albicans* biofilms [70]. A thionin-like peptide from *Capsicum annuum* fruits exhibited synergy with fluconazole against *Fusarium solani* [71], as well as against various *Candida* species [72]. Further, several studies have identified synergy between specific fragments of AMPs and traditional antibiotics. For example, fragments of the hevein-like peptide WAMP-2 from *Triticum kiharae* acted synergistically with tebuconazole to target plant pathogenic fungi such as *Fusarium* spp., *Alternaria* spp., *B. sorokiniana*, and *Parastagonospora nodorum* [73,74]. Similarly, fragments of NCR defensin-like peptides from legume nodules showed synergy with fluconazole, inhibiting the growth of various *Candida* species [75].

## 4. Materials and Methods

### 4.1. Chemical Synthesis of Short Defensin-Derived Peptides

Five peptide fragments encompassing the *γ*-core sequences of three *T. elongatum* and two *T. kiharae* DEFLs were chemically synthesized using the Fmoc method (Elabscience Biotechnology Inc., Wuhan, China). The synthetic peptides were purified by RP-HPLC. Their identity to the required sequences was confirmed by matrix-assisted laser desorption/ionization time-of-flight (MALDI-TOF) mass spectrometric analysis using an Ultraflex MALDI-TOF mass spectrometer (Bruker Daltonics, Bremen, Germany) in a linear or reflector positive ion mode using *α*-cyano-4-hydroxycinnamic acid as a matrix.

### 4.2. In Silico Characterization of the Synthetic Peptides

The Peptide Search Tool was used to screen for UniProt entries with the synthesized peptides as queries [33]. All alignments were constructed using Vector NTI Advance 9 software (Invitrogen, Waltham, MA, USA). The physicochemical characteristics of the synthesized peptides, such as molecular weight, net charge at pH 7, pI, GRAVY index, and aliphatic index, were computed using the ExPASy ProtParam tool [76]. Hydrophobic moment μH was calculated with HeliQuest [77]. Boman index, hydrophobicity and ratio of hydrophobic residues were calculated using APD3 [78]. Prediction of antimicrobial properties was carried out with CAMPR4 [79]. The 3D structure of the synthetic peptides was de novo modeled using the PEP-FOLD4 program [35]. The best representative models were considered those that had the lowest sOPEP values provided by PEP-FOLD4.

### 4.3. Antimicrobial Assays

#### 4.3.1. Antifungal Activity Assays

The antifungal activity of peptides was tested against nine fungal strains from All-Russian Collection of Microorganisms (VKM, Pushchino, Russia): *Fusarium culmorum* VKM F-2303, *F. oxysporum* VKM F-137, *F. solani* VKM F-142, *F. verticillioides* VKM F-670, *Aspergillus unguis* VKM F-1754, *Bipolaris sorokiniana* VKM F-4006, *Botrytis cinerea* VKM F-4549, *Rhizoctonia solani* VKM F-895, and *Penicillium gladioli* VKM F-2088. Cultivation of fungi and spore isolation were performed as described [62]. The antifungal activity of the peptides was assessed by measuring the optical density of the spore suspension in 96-well microplates in the presence of the peptide relative to the control. Each well contained 90 μL of the spore suspension in half-strength potato dextrose broth (2000–3000 spores per 100 μL) and 10 μL of peptide aqueous solutions (final concentrations ranging from 5 to 300 μM). The spore concentration in the suspension was determined using a Goryaev chamber, and the optical density was recorded at 595 nm on a FilterMax F5 Multi-Mode Microplate Reader (Molecular Devices, San Jose, CA, USA) after 48 h of incubation. The IC_50_ values, the peptide concentrations required to achieve 50% inhibition of pathogen growth, were determined from dose–response curves. The IC_50_ values are presented as mean ± standard deviations.

#### 4.3.2. Antimicrobial Activity Assays Against Bacteria and Yeasts

The synthetic peptides were evaluated for their antimicrobial effectiveness against the yeasts *Cryptococcus neoformans* VKM Y-2755, *Candida albicans* VKM Y-2994 and *C. tropicalis* VKM Y-2771 and the bacteria *Pectobacterium carotovorum* subsp. *carotovorum* VKM B-1247, *Pseudomonas savastanoi* pv. *savastanoi* VKM B-1546 and *Clavibacter michiganensis* subsp. *michiganensis* VKM Ac-1403. Yeasts and bacterial cultures were cultivated as described [62]. The antimicrobial activity of peptides against yeasts and bacteria was tested using 96-well plates for immunoassays. In each well, 10 μL of the tested peptide aqueous solution (with final concentrations ranging from 0.5 to 300 μM), 10 μL of the microbial suspension diluted with water at a ratio of 1:20, and 80 μL of medium were combined, then incubated at 30 °C for 24 h. Yeasts were grown on the YPD-P medium, which contained (g/L) glucose—10, peptone—5, yeast extract—4. *P. carotovorum* and *C. michiganensis* were grown on a modified YPD-P medium (g/L): glucose—5, peptone—10, yeast extract—5. For the experiments with *P. savastanoi*, the medium did not contain glucose, and the concentrations of peptone and yeast extract were 20 g/L and 10 g/L, respectively. The plates were incubated at 700 rpm for 24 h at 30 °C. The optical density of the pathogen cultures was measured using an Efos 9305 spectrophotometer (Sapphire, Moscow, Russia) at a wavelength of 594 nm. Antimicrobial tests for each pathogen were performed in three replicates for each treatment. The antimicrobial activity was expressed in IC_50_ values.

#### 4.3.3. Synergistic Interactions Assays

The ability of synthetic peptides to enhance the inhibitory effect in co-application was assessed against the fungus *F. oxysporum* and the bacterium *C. michiganensis*. The study of antimicrobial activity in microtiter plates was carried out as described above with the following modification: 5 µL of each peptide aqueous solution in different combinations of concentrations was added to the wells. For the experiments with *F. oxysporum*, the final peptide concentrations of 0–150 µM were used; for the experiments with *C. michiganensis*, the peptide concentrations were 0–100 μM. Experiments were performed in three replicates for each treatment. The pathogen growth inhibition (%) was calculated relative to the control (growth without peptide addition).

To assess the synergy between two *γ*-core peptides, Limpel’s criterion Ee < Er [80] was calculated using the following Formula (1):

Ee,% = (X + Y) − XY/100 < Er,% (at *p* ≤ 0.05), (1)

where Ee represents the expected additive effect from combining both peptides; X and Y indicate the percentage of pathogen growth inhibition by each peptide individually; and Er denotes the observed inhibition in % when both peptides are applied simultaneously.

### 4.4. Statistical Analysis

Mean values, standard deviations (SD) and the significance of differences (*p* ≤ 0.05) of the means between treatments and controls (*t*-test for independent variables) were determined with the Microsoft Excel program (Microsoft, Redmond, WA, USA).

### 4.5. Propidium Iodide Staining of Pathogen Cells

Three yeast pathogens, *C. albicans*, *C. tropicalis* and *C. neoformans*, were chosen for analysis. The pathogen cells were grown on the YPD-P medium at 28 °C for 24 h, 24 h, and 48 h, respectively. The cultures were diluted 1:20 with water and incubated with the peptide at a concentration of 100 µM at 30 °C for 1.5 h. To stain the cells, propidium iodide (Sigma-Aldrich, St. Louis, MO, USA) was added to the medium to reach a final concentration of 3 µg/mL. After a brief (1–2 min) incubation at room temperature, the samples were analyzed on a NovoCyte Flow cytometer (Agilent, Santa Clara, CA, USA) using 488 nm for excitation and 572/28 nm for emission (a total of 100,000 cells were counted for each sample). The samples without peptides were used as a control. All experiments were performed in triplicate.

## 5. Conclusions

In conclusion, our study demonstrates that chemically synthesized *γ*-core peptides derived from wheatgrass defensins exhibit potent antimicrobial activity against both plant and opportunistic human pathogens, and some of them act synergistically with each other, thus reducing the effective dose for pathogen inhibition. It was also shown that membrane permeabilization is one of the mechanisms of at least some of the peptides’ action. The sequences of wheatgrass defensin-derived *γ*-cores are widely distributed among the defensins of Poaceae plants, suggesting their evolutionary conservation and indicating the functional significance of these regions of the molecule. Our work also highlights the importance of positive charge in the antimicrobial activity of the wheatgrass *γ*-core peptides since the most active peptide, Tedef1, has the highest net charge of +5 compared to other peptides tested. The role of the peptide’s *β*-hairpin fold in its exceptional antimicrobial potency cannot be underestimated. Our findings suggest that even small, single-amino acid modifications in the *γ*-core peptide sequences can significantly influence their effectiveness in fighting microbes. Our results emphasize the potential of the synthetic *γ*-core peptides as promising templates for the development of plant-based biopesticides. Further analysis of the stability and cytotoxicity of the short peptide molecules, along with subsequent structural modifications to enhance practical properties, such as antimicrobial potency, molecular size, stability, and reduced potential toxicity to plant and human cells, will ultimately facilitate the development of new, safe, and effective peptide-based antimicrobials for plant protection and medicine.

## Figures and Tables

**Table 1:** Sequences of synthetic peptides.

Synthetic Peptide	Peptide Origin	Amino Acid Sequence	Length, aa	Molecular Weight, Da
Tedef1	TeDEFL1-16	GKCRGFRRRCFCTTHC	16	1931.30
Tedef2	TeDEFL1-32	GYCKIRRCTCQREC	14	1719.05
DEFL1-32_55–68_	TkDEFL1-32	GYCKFRRCTCQREC	14	1753.06
Tedef4	TeDEFL1-12	GNCDGPLRRCKCIRQC	16	1822.17
DEFL1-12_62–77_	TkDEFL1-12	GNCDGPFRRCKCIRQC	16	1856.19

**Table 2:** Physicochemical properties of synthetic peptides.

Peptide	Net Charge at pH 7	pI	Aliphatic Index	Boman Index	Ratio of Hydrophobic Residues, %	μH	GRAVY Index	AMP Prediction
Tedef1	+5	9.89	0.00	3.87	38	0.195	−0.731	AMP
Tedef2	+3	8.94	27.86	3.88	36	0.109	−0.879	AMP
DEFL1-32_55–68_	+3	8.94	0.00	4.02	36	0.109	−1.000	AMP
Tedef4	+3	8.98	48.75	3.39	38	0.318	−0.750	AMP
DEFL1-12_62–77_	+3	8.98	24.38	3.51	38	0.317	−0.812	AMP

**Table 3:** Antimicrobial activity of the *γ*-core motif peptides *.

Pathogen	IC_50_, μM				
	Tedef1	Tedef2	DEFL1-32_55–68_	Tedef4	DEFL1-12_62–77_
*Fusarium culmorum*	12.5 ± 0.2	44.4 ± 6.7	51.5 ± 4.1	155.6 ± 68.1	92.3 ± 9.5
*Fusarium oxysporum*	34.3 ± 5.7	56.3 ± 5.3	151.9 ± 12.6	–	–
*Fusarium solani*	26.8 ± 2.3	–	201.4 ± 17.1	–	–
*Fusarium verticillioides*	16.9 ± 0.8	73.5 ± 10.7	79.5 ± 9.2	103.8 ± 9.1	147.7 ± 16.3
*Aspergillus unguis*	40.3 ± 2.3	42.5 ± 2.9	21.2 ± 8.2	2.1 ± 0.0	2.0 ± 0.0
*Penicillium gladioli*	67.6 ± 0.2	67.7 ± 0.4	144.6 ± 51.4	125.5 ± 20.8	71.0 ± 39.7
*Bipolaris sorokiniana*	97.9 ± 4.1	119.1 ± 16.3	146.5 ± 6.4	145.1 ± 19.6	182.7 ± 25.0
*Botrytis cinerea*	107.6 ± 5.8	–	–	–	–
*Rhizoctonia solani*	99.7 ± 6.8	–	–	–	–
*Cryptococcus neoformans*	10.6 ± 0.5	9.9 ± 0.2	12.5 ± 0.5	29.8 ± 1.3	28.6 ± 7.5
*Candida albicans*	7.3 ± 1.8	–	–	–	–
*Candida tropicalis*	5.9 ± 0.8	26.5 ± 0.8	7.8 ± 1.0	100.2 ± 7.4	96.0 ± 9.9
*Clavibacter michiganensis*	29.4 ± 1.0	168.0 ± 0.3	–	182.3 ± 0.6	–
*Pseudomonas savastanoi*	45.2 ± 0.2	226.4 ± 0.6	245.7 ± 14.8	–	–
*Pectobacterium carotovorum*	–	–	–	–	–

* Mean values ± SD are presented; – indicates no activity at peptide concentrations below 300 μM.

**Table 4:** Observed inhibitory effect (Er, %) on *F. oxysporum* growth of the Tedef2/Tedef4 pair compared to the calculated additive inhibitory effect (Ee, %). Er values of peptide concentration combinations for which Ee < Er are shown in bold. Er values of peptide concentration combinations for which Ee < Er is fulfilled at *p* ≤ 0.05 are framed.

Concentration of Tedef2, μM	Concentration of Tedef4, μM														
	0	5		10		25		50		75		100		150	
	Er	Er	Ee	Er	Ee	Er	Ee	Er	Ee	Er	Ee	Er	Ee	Er	Ee
0		0		0		1.1		0		20.6		10.9		10.3	
5	0	**12.5**	0	**15.0**	0	**16.8**	1.1	**23.3**	0	**24.3**	20.6	**23.8**	10.9	**27.5**	7.3
10	0	**11.1**	0	**13.2**	0	**16.1**	1.1	**21.8**	0	**23.3**	20.6	**22.4**	10.9	**30.4**	7.3
25	6.3	**18.4**	6.3	**22.9**	6.3	**23.6**	7.3	**24.7**	6.3	**27.2**	25.5	**26.1**	16.5	**30.2**	13.1
50	44.9	20.6	44.9	23.4	44.9	22.9	45.5	**61.5**	44.9	**76.4**	56.2	**78.9**	50.9	**77.3**	48.9
75	51.7	**77.5**	51.7	**81.2**	51.7	**78.7**	52.2	**77.8**	51.7	**82.5**	61.6	**76.6**	57.0	**78.0**	55.2
100	60.8	**79.6**	60.8	**77.5**	60.8	**80.1**	61.2	**80.9**	60.8	**78.0**	68.9	**79.1**	65.1	**79.4**	63.7

**Table 5:** Observed inhibitory effect (Er, %) on *C. michiganensis* of the Tedef2/Tedef4 pair compared to the calculated additive inhibitory effect (Ee, %). Er values of peptide concentration combinations for which Ee < Er are shown in bold.

Concentration of Tedfe2, μM	Concentration of Tedef4, μM										
	0	10		25		50		75		100	
	Er	Er	Ee	Er	Ee	Er	Ee	Er	Ee	Er	Ee
0		5.4		16.3		17.3		26.0		28.8	
10	2.5	6.4	7.8	12.0	18.4	12.9	19.4	17.6	27.8	18.5	30.6
25	13.2	13.6	17.8	13.2	27.3	15.5	28.2	19.6	35.7	20.7	38.1
50	16.7	17.2	21.2	19.9	30.3	16.7	31.2	20.6	38.3	21.6	40.7
75	27.4	27.7	31.3	31.2	39.2	33.9	40.0	36.1	46.2	36.2	48.3
100	28.2	**33.1**	32.0	33.6	39.9	36.6	40.6	40.5	46.8	41.2	48.8

## Data Availability

The original contributions presented in this study are included in the article. Further inquiries can be directed to the corresponding authors.

## Abbreviations

The following abbreviations are used in this manuscript:

AMP	Antimicrobial Peptide
DEFL	Defensin-like Peptide
pI	Isoelectric point
μH	Hydrophobic moment
GRAVY	Grand Average of Hydropathy
IC_50_	Peptide concentration required for 50% inhibition of the pathogen growth
SD	Standard Deviation
PI	Propidium Iodide
